# Heartache and Heartbreak: An Observational and Mendelian Randomization Study

**DOI:** 10.5334/gh.1302

**Published:** 2024-02-16

**Authors:** Dihui Cai, Mengming Xia, Xuhui Chen, Kunimasa Yagi, Liang Xu, Bingyu Wang, Yanyi Wang, Yujie Zhou, Jianhui Liu

**Affiliations:** 1 Department of Cardiology, Ningbo Medical Center of Lihuili Hospital, Ningbo, Zhejiang, China; 2 Department of Pharmacy, Ningbo Medical Center of Lihuili Hospital, Ningbo, Zhejiang, China; 3 School of Medicine, Kanazawa Medical University, Kanazawa, Ishikawa, Japan; 4 School of Laboratory Medicine and Life Sciences, Wenzhou Medial University, Wenzhou, Zhejiang, China; 5 Health Science Center, Ningbo University, Ningbo, Zhejiang, China

**Keywords:** Mendelian randomization, depression, cardiovascular diseases

## Abstract

**Background:**

Depression has a significant effect on cardiovascular disease (CVD), but uncertainties persist regarding which modifiable risk factors mediate the causal effects. We aim to determine whether depression is causally linked to CVD and which modifiable risk factors play potential mediating roles.

**Methods:**

We used a two-sample Mendelian randomization (MR) approach and NHANES 2007–2018 data to estimate the effects of depression on various CVD cases and investigated 28 potential mediators of the association between depression and CVD.

**Results:**

The results of our MR analysis indicated that genetically determined depression was associated with increased risk of several CVD, including coronary heart disease (odds ratio (OR) = 1.14; 95% confidence interval (CI): 1.05,1.22), myocardial infarction (OR = 1.19; 95% CI, 1.09,1.31), atrial fibrillation (OR = 1.14; 95% CI, 1.06,1.22), and stroke (OR = 1.13; 95% CI, 1.05,1.22). However, there was no causal association between depression and heart failure. Four out of 28 cardiometabolic risk factors, including hyperlipidemia, hypertension, diabetes, and prescription opioid use, were identified as mediators of the association between depression and various CVDs. Observational association analyses from NHANES data yielded consistent results.

**Conclusion:**

Our findings demonstrated that depression has a causal detrimental effect on various CVDs. Four causal mediators (hyperlipidemia, hypertension, diabetes, and prescription opioid use) were screened to explain the causal effect. Implementing targeted management strategies for these risk factors may be warranted to mitigate the public health burden of CVD among individuals with depression.

## Introduction

Cardiovascular diseases (CVDs) remain the primary reason for global mortality due to chronic illnesses [1,2]. They pose a substantial and persistent challenge to healthcare systems [3]. In the year 2020, CVDs were responsible for the deaths of approximately 19 million individuals across the globe, marking a concerning 18.7% rise over the span of a decade [4]. A recently released report emphasized the need for medical professionals to be aware of the connection between psychiatric traits related to stress and CVDs [5].

Depression is a prevalent mental illness on a global scale, impacting more than 264 million individuals in the year 2017 [6]. In individuals with depression, there is a frequent coexistence of CVD. Mounting observational studies have consistently demonstrated a correlation between depression and CVD, including coronary heart disease (CHD) [7,8], myocardial infarction (MI) [8,9], stroke [10,11], heart failure (HF) [9,12], and atrial fibrillation (AF) [13]. Given the quality and duration of life for people with depression, it is important to understand what drives these associations between depression and CVD, since our current understanding is limited.

Mendelian randomization (MR) analysis is an epidemiological tool employing genetic variants as instrumental variables (IVs) for a trait to confirm a causal association between the trait and an outcome [14]. According to the random distribution of genetic variants from parents to offspring, MR effectively mitigates potential confounding factors and reverse causation biases that often plague observational studies, thereby strengthening causal inference [15].

To investigate the potential causal relationship between depression and various CVDs, as well as the mediating effect of cardiometabolic risk factors on these associations, this study used data from the National Health and Nutrition Examination Survey (NHANES) database and a two-sample MR analysis. Mediation analysis and a strict screening process were conducted to further investigate the mediating effect of cardiometabolic risk factors for these relationships.

## Material and Methods

### Study Design

**Figure 1:**
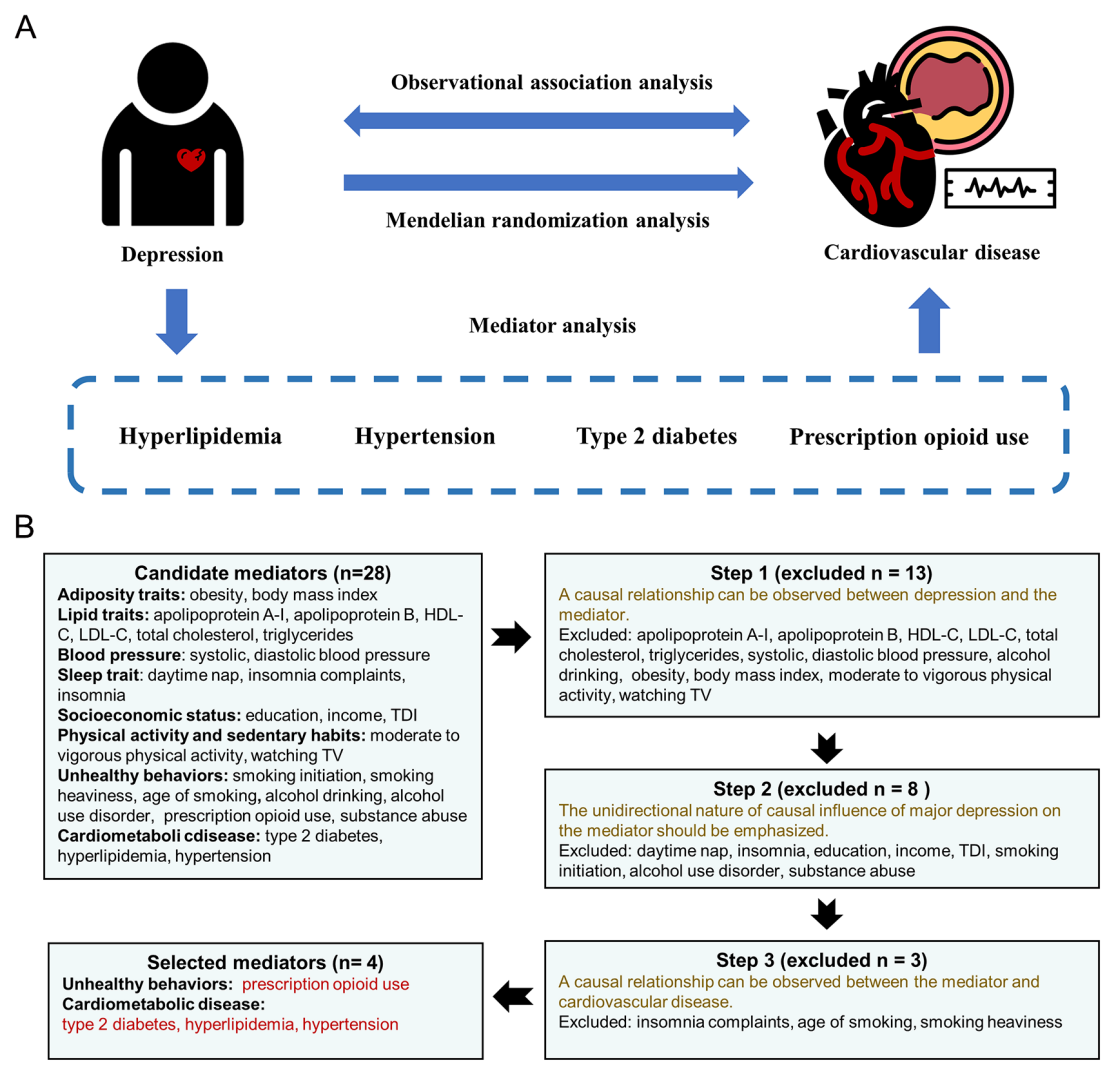
**A.** Study design. **B.** Mediator screening process. HDL-C, high-density lipoprotein cholesterol; LDL-C, low-density lipoprotein cholesterol; TDI, Townsend deprivation index; TV, television.

Figure 1A presents an outline of the study design. The study used NHANES data to analyze the association between depression and various CVDs and modifiable cardiometabolic risk factors. Subsequently, we employed two-sample MR analyses to investigate the causal relationship of genetic susceptibility to depression on the risk of various CVDs and modifiable cardiometabolic risk factors, and assessed the degree of mediation effect using two-step MR. A rigorous screening process was conducted to identify potential mediators between depression and CVD based on the criteria shown in Figure 1B. The MR study was conducted in accordance with the STROBE-MR guidelines.

Due to the data in the study being retrieved from the publicly available NHANES database and genome-wide association studies (GWASs), the necessity for obtaining approval from the ethical committee was exempted.

### Data Sources

#### Data sources of observational study

The NHANES dataset is a sample that represents the population of the United States that is not institutionalized, and it is collected by the National Center for Health Statistics (NCHS). This initiative annually surveys approximately 5,000 nationally representative individuals through a stratified multistage sampling methodology. The observational research section in our study utilized data from the NHANES database spanning from 2007 to 2018.

##### Study population in NHANES database

In the initial sample, we identified a total of 59,842 participants. Of these, we excluded participants without data on the incomplete depression questionnaires (n = 28382), CVD outcomes (n = 1892), and confounding factor information (n = 15573). Finally, 9645 subjects aged >20 years were enrolled for further analysis (Figure S1).

##### Assessment of depression, CVD outcomes, and covariates

For the NHANES participants, the 9-item Patient Health Questionnaire (PHQ–9) was utilized as a depression screening tool to assess depression [16]. The overall score spans from 0 to 27, with a score of 10 or higher indicating the presence of depression [17].

CVD outcomes comprised the presence of angina, coronary heart disease, heart attack, congestive heart failure, or stroke as reported diagnoses. The associated survey offered individual-level interview information to evaluate CVD outcomes. Skilled interviewers utilized a computer-assisted personal interview system to inquire about these matters in interviewee’s residence.

Demographic characteristics information was obtained from household interviews, including gender, age, race, ratio of family income to poverty, and education level. Information on the history of drinking, smoking, hypertension, diabetes, and dyslipidemia was collected from the health questionnaire. Examination data information included diastolic blood pressure (DBP, mmHg), systolic blood pressure (SBP, mmHg), and body mass index (BMI, kg/m2). Standardized methods were used to determine the values of total cholesterol (TC, mmol/L), low-density lipoprotein cholesterol (LDL-C, mmol/L), high-density lipoprotein cholesterol (HDL-C, mmol/L), and triglycerides (TG, mmol/L).

#### Data sources in MR analysis

Throughout the MR study, we utilized summary-level data from publicly available GWASs to obtain data sources for exposures, outcomes, and mediators in participants of European ancestry. Table S2 lists a detailed description of all the GWAS datasets associated with all exposure, outcomes, and mediators.

##### Exposures

Genetic IVs utilized to represent depression were derived from the largest published GWAS meta-analysis [18] with a sample of 500,199 European ancestry participants, including the Psychiatric Genomic Consortium (PGC) (43,204 cases, 95,680 controls) and UK biobank (127,552 cases, 233,763 controls) after the exclusion of 23andMe participants due to insufficient single nucleotide polymorphisms (SNPs) data.

SNPs have been utilized as IVs to serve as proxies for each exposure in the study. The entire procedure adhered to the three primary assumptions. (a) IVs exhibit a strong correlation with exposure factors. (b) IVs do not demonstrate any association with confounding factors. (c) The influence of IVs on the risk of outcomes is just through exposure [19]. Forty-nine independent genome-wide significant (P < 5 × 10^–8^) SNPs were chosen as the main genetic instruments for investigating the causal relationship of depression with various CVDs. The linkage disequilibrium analyses were conducted to assess the presence of linkage disequilibrium (r^2^ < 0.001; distance threshold, 10 000 kb).

##### Mediators

Based on published studies, depression was associated with several cardiometabolic risk factors, we have chosen 28 potential modifiable cardiometabolic mediators, such as adiposity traits [20] (BMI obesity), blood lipid [21,22] (hyperlipidemia, HDL-C, LDL-C, TC, TG), blood pressure [23] (hypertension, SDP, DBP), type–2 diabetes [22,24], smoking status [25], unhealthy habits [26,27] (the initiation of smoking, smoking heaviness, the age of smoking, the consumption of alcohol, alcohol use disorder, prescription opioid use, substance abuse), physical activity [28], sedentary habits [29] (the time of watching TV and moderate to vigorous physical activity), sleep trait [30] (insomnia, daytime nap), and socioeconomic status [31,32] (education, income, Townsend deprivation index).

Subsequently, we conducted a screening process to identify potential mediators of the connection between depression and CVD based on the subsequent criteria: (1) A causal relationship can be observed between depression and the mediator; (2) The unidirectional nature of the causal influence of depression on the mediator should be emphasized, meaning that depression should only have an impact on the mediator and not vice versa. The presence of bidirectionality between exposure and mediator can potentially impact the validity of results [33]; (3) A causal relationship can be observed between the mediator and cardiovascular disease. Figure 1B depicts the comprehensive process for selecting a mediator. Four cardiometabolic risk factors ultimately satisfied all the established criteria. Figure S2 showed the calculation process of proportion mediated of potential mediators.

##### Outcomes

Related GWAS datasets and FinnGen consortiums, having no overlap with the samples of depression GWAS, were used to obtain data for various CVDs. The FinnGen Study is a comprehensive nationwide GWAS meta-analysis conducted in Finland. In addition, to minimize bias arising from population stratification, the study exclusively employed GWAS datasets comprising individuals of European ancestry.

### Statistical analysis

#### Statistical analysis of the observational study

Mean (standard deviation) is used to present continuous variables, whereas numbers (percentage) are used to present categorical variables. T-tests were used to compare the demographic and health characteristics of participants with and without CVD for continuous variables, while the chi-squared test was employed for categorical variables.

A stepwise regression model was employed to establish the correlation between depression and different CVDs by utilizing multivariate logistic regression models. In Model 1, we computed the odds ratio (OR) value using univariate logistic regression. In Model 2, we adjusted for potential confounding factors, including gender, age, race, poverty, and education level. In Model 3, a comprehensive adjustment was implemented based on the adjustments made in Model 2, for smoking, drinking, hypertension, diabetes, dyslipidemia, TC, TG, HDL-C, LDL-C, SBP, DBP, and BMI. In addition, Multivariate logistic regression models, after adjusting for gender, age, and race, were employed to determine the relationship between depression and various confounding factors, including smoking, drinking, hypertension, diabetes, dyslipidemia, education, income, and BMI. Statistical significance was determined using a two-sided approach for all tests. To address the issue of multiple testing, Bonferroni correction was used. Corrected p value = 0.05/6 = 0.0083 was used for association analysis of depression on CVD, and corrected p value = 0.05/8 = 0.0063 for association analysis of depression on CVD risk factors.

#### Statistical analyses of MR study

The MR study employed R software (version 4.2.0) and various R packages including “TwoSampleMR”, “MR-PRESSO”, and “meta” for statistical analysis. Multiple MR approaches were employed to assess causal relationships. All methods are predicated on distinct assumptions regarding the efficacy of IVs.

The main method used in MR analyses was the application of the inverse-variance weighted (IVW) approach, which consistently produced reliable results when all IVs were found to be valid [34]. We also used MR-pleiotropy residual sum and outlier (MR-PRESSO) to observe and correct for outliers in the IVW linear regression [35]. MR-Egger [36], weighted median [37], and MR-PRESSO approaches were additionally employed as secondary analysis methods. We combined the IVW outcomes for CVD from FinnGen and related GWAS utilizing meta-analysis.

To assess the reliability of the findings, we conducted a sequence of sensitivity analyses. The assessment of heterogeneity of the utilized SNPs was conducted through the application of Cochrane’s Q test, and if significant heterogeneity was present (P < 0.05), we used the multiplicative random effects IVW method as the primary approach to obtain a robust estimate. Additionally, the MR-Egger regression method was utilized to assess the existence of directional pleiotropy among the IVs by examining its intercept. Conditional F-statistics were employed to assess the validity of instruments, with an F > 10 indicating the sufficiently strong association between the IVs and exposure and avoids a weak instrument bias (Table S1).

Bonferroni correction (corrected p = 0.05/10 = 0.005) was used. P values of IVW estimates ranging from 0.005 to 0.05 without pleiotropy (P > 0.05) were interpreted as indicative of a potential causal relationship between the depression and CVD outcomes, and P values < 0.005 without pleiotropy were deemed to signifying a significant causal relationship.

## Results

### Results of observational study from NHANES database

#### Baseline characteristics

Table 1 displays the characteristics of the 9,645 qualified participants. The mean (SD) age of all participants was 50.58 (17.3) years, with 4,718 (48.9%) of them being male. The participants were categorized into two groups: the non-CVD group (n = 8,577) and the CVD group (n = 1,068). Individuals in the CVD group were characterized by their higher age compared to those in the non-CVD group. Additionally, they exhibited a greater likelihood of being male or identifying as non-Hispanic Caucasian. Moreover, they had a lower level of education, elevated poverty index, higher BMI, increased smoking prevalence, and a greater occurrence of hypertension, dyslipidemia, and diabetes (P < 0.05) (Table 1). The lower total cholesterol and LDL-C levels in the CVD group may be attributed to the administration of lipid-lowering medication in these patients.

**Table 1:** The characteristics of included participants.


	TOTAL	NON-CVD (n = 8577, 88.93%)	CVD (n = 1068, 11.07%)	P-VALUE

Age, years, mean (SD)	50.6 (17.3)	48.7 (16.9)	66.10 (12.2)	<0.001

Gender, Male n (%)	4718 (48.9%)	4104 (47.9%)	614 (57.5%)	<0.001

Race, n (%)				<0.001

Mexican American	1286 (13.3%)	1195 (13.9%)	91 (8.5%)	

Other Hispanic	968 (10.0%)	889 (10.4%)	79 (7.4%)	

Non-Hispanic White	4329 (44.9%)	3719 (43.4%)	610 (57.1%)	

Non-Hispanic Black	1916 (19.9%)	1690 (19.7%)	226 (21.2%)	

Other Race – Including Multi-Racial	1146 (11.9%)	1084 (12.6%)	62 (5.8%)	

Education level, n (%)				<0.001

High school and below	4151 (43.0%)	3567 (41.6%)	584 (54.7%)	

Above high school	5494 (57.0%)	5010 (58.4%)	484 (45.3%)	

Ratio of family income to poverty, ratio, mean (SD)	2.6 (1.6)	2.6 (1.7)	2.3 (1.5)	<0.001

PHQ-9, points, n (%)	809 (8.4%)	640 (7.5%)	169 (15.8%)	<0.001

Smoking, n (%)	4291 (44.5%)	3643 (42.5%)	648 (60.7%)	<0.001

Drinking, n (%)	7325 (76.0%)	6524 (76.1%)	801 (75.0%)	0.443

History of dyslipidemia, n (%)	3542 (36.7%)	2851 (33.2%)	691 (64.7%)	<0.001

History of hypertension, n (%)	3675 (38.1%)	2888 (33.7%)	787 (73.7%)	<0.001

History of diabetes, n (%)	1359 (14.1%)	993 (11.6%)	366 (34.3%)	<0.001

SBP, mmHg, mean (SD)	124.2 (18.5)	123.3 (18.0)	131.3 (21.1)	<0.001

DBP, mmHg, mean (SD)	70.3 (11.7)	70.5 (11.5)	68.0 (13.1)	<0.001

BMI, kg/m2, mean (SD)	29.2 (6.9)	29.1 (6.8)	30.5 (7.3)	<0.001

TG, mmol/L, mean (SD)	1.3 (0.7)	1.3 (0.7)	1.5 (0.8)	<0.001

TC, mmol/L, mean (SD)	4.9 (1.1)	5.0 (1.0)	4.6 (1.1)	<0.001

HDL, mmol/L, mean (SD)	1.4 (0.4)	1.4 (0.4)	1.3 (0.4)	<0.001

LDL, mmol/L, mean (SD)	2.9 (0.9)	3.0 (0.9)	2.6 (1.0)	<0.001


CVD, cardiovascular disease; PHQ-9, 9-item Patient Health Questionnaire; TG, triglyceride; TC, total cholesterol; HDL-C, high-density lipoprotein cholesterol; LDL-C, low-density lipoprotein cholesterol; BMI, body mass index; SBP, systolic blood pressure; DBP, diastolic blood pressure.

#### Multiple logistic regression analysis between depression and CVD

Model 2 and Model 3, which have been adjusted for demographic variables and various CVD risk factors, respectively, demonstrate the association between depression and CVD through a stepwise regression analysis. After the adjustment for potential confounding factors (gender, age, race, education, poverty index, hypertension, diabetes, dyslipidemia, smoking, drinking, blood pressure, BMI, TC, TG, HDL, and LDL), depression was significantly associated with higher odds of CVD outcomes (OR = 2.07; 95% CI, 1.55–2.76; P < 0.001), heart attack (OR = 1.77; 95% CI, 1.22–2.56; P = 0.004), angina pectoris (OR = 2.17; 95% CI, 1.37–3.43; P = 0.001), HF (OR = 2.45; 95% CI, 1.51–3.98; P < 0.001), and stroke (OR = 2.06; 95% CI, 1.21–3.53; P = 0.010), and suggestively associated with CHD (OR = 1.73; 95% CI, 1.12–2.69; P = 0.016) (Table 2).

**Table 2:** Logistic regression associations of depression with CVD in adults.


VARIABLE	MODEL 1		MODEL 2		MODEL 3	
		
	OR (95% CI)	*P* VALUE	OR (95% CI)	*P* VALUE	OR (95% CI)	*P* VALUE

Cardiovascular disease	2.36(1.83–3.05)	<0.001	2.60(1.95–3.46)	<0.001	2.07(1.55–2.76)	<0.001

Coronary heart disease	1.71(1.14–2.56)	0.011	2.14(1.44–3.18)	<0.001	1.73(1.12–2.69)	0.016

Heart attack	2.13(1.52–3.00)	<0.001	2.21(1.52–3.21)	<0.001	1.77(1.22–2.56)	0.004

Angina pectoris	2.82(1.95–4.08)	<0.001	2.90(1.97–4.28)	<0.001	2.17(1.37–3.43)	0.001

Heart failure	3.43(2.26–5.20)	<0.001	3.10(1.91–5.03)	<0.001	2.45(1.51–3.98)	<0.001

Stroke	2.75(1.72–4.40)	<0.001	2.44(1.41–4.21)	0.002	2.06(1.21–3.53)	0.010


Model 1 was univariate logistic regression model; Model 2 was adjusted as age, race, gender, education, and ratio of family income to poverty; Model 3 was adjusted as age, race, gender, education, ratio of family income to poverty, smoking, drinking, dyslipidemia, hypertension, diabetes, diastolic pressure, systolic pressure, TG, TC, HDL-C, LDL-C and BMI based on stepwise regression model. OR, odds ratio; 95% CI, 95% confidence interval; TG, triglyceride; TC, total cholesterol; HDL-C, high-density lipoprotein cholesterol; LDL-C, low-density lipoprotein cholesterol.

#### Multiple logistic regression analysis between depression and CVD risk factors

We also estimated the association of depression and possible CVD risk factors (Table 3). The results of weighted multivariate logistic regression (adjusted for age, race, and gender) indicated that depression was significantly associated with higher odds of various CVD risk factors, including smoking (OR = 2.60; 95% CI, 2.08–3.23; P < 0.001), hypertension (OR = 1.84; 95% CI, 1.46–2.32; P < 0.001), diabetes (OR = 2.15; 95% CI, 1.62–2.85; P < 0.001), dyslipidemia (OR = 1.54; 95% CI, 1.22–1.93; P < 0.001), BMI (β = 1.97; 95% CI, 1.39, 2.55; P < 0.001). Meanwhile, depression was significantly associated with lower level of education (OR = 0.48; 95% CI, 0.38–0.59; P < 0.001) and income (β = –0.95; 95% CI, –1.10, –0.81; P < 0.001). As shown in Table 3, these risk factors were significantly associated with CVD outcomes.

**Table 3:** The association of depression and CVD risk factors in adults.


VARIABLE	DEPRESSION AND CVD RISK FACTORS			CVD RISK FACTORS AND CVD		
	
	β (95% CI)	OR (95% CI)	*P* VALUE	β (95% CI)	OR (95% CI)	*P* VALUE

Smoking	0.95(0.73,1.17)	2.60(2.08–3.23)	<0.001	0.52(0.33,0.71)	1.68(1.39,2.03)	<0.001

Drinking	0.19(–0.02,0.4)	1.21(0.98–1.49)	0.081	0.06(–0.15,0.27)	1.06(0.86,1.31)	0.582

Hypertension	0.61(0.38,0.84)	1.84(1.46–2.32)	<0.001	0.94(0.74,1.15)	2.16(1.75,2.65)	<0.001

Diabetes	0.77(0.49,1.05)	2.15(1.62–2.85)	<0.001	0.90(0.66,1.14)	2.57(2.10,3.15)	<0.001

Dyslipidemia	0.43(0.20,0.66)	1.54(1.22–1.93)	<0.001	0.77(0.56,0.98)	2.45(1.93,3.12)	<0.001

Education	–0.74(–0.96,–0.52)	0.48(0.38–0.59)	<0.001	–0.35(–0.53,–0.16)	0.71(0.59,0.85)	<0.001

Income	–0.95(–1.10,–0.81)		<0.001	–0.19(–0.25,–0.13)	0.83(0.78,0.88)	<0.001

BMI	1.97(1.39,2.55)		<0.001	0.05(0.03,0.06)	1.05(1.03,1.06)	<0.001


Logistic regression model was adjusted as age, race, gender. OR, odds ratio; 95% CI, 95% confidence interval; CVD, cardiovascular disease; BMI, body mass index.

### Result of Mendelian randomization study

#### Effects of depression on CVD

**Figure 2:**
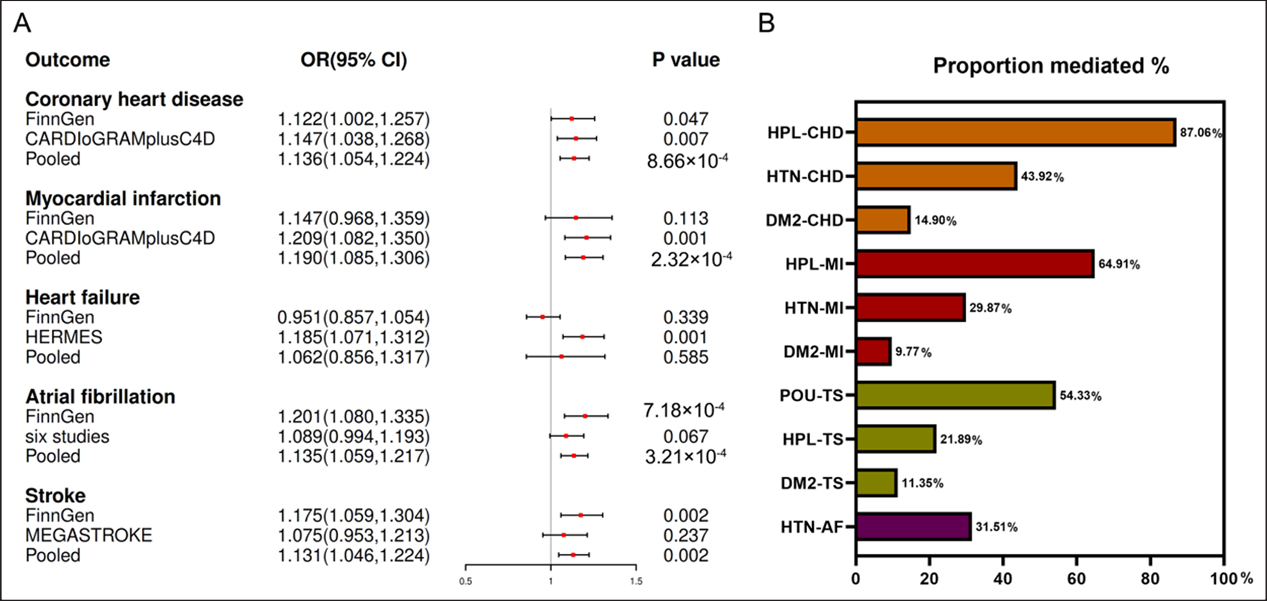
**A.** Mendelian randomization (MR) estimates of the causal associations of depression on cardiovascular diseases. **B.** MR estimates of proportions mediated by mediators in the causal association between depression on cardiovascular diseases. OR, odds ratio; CI, confidence interval; HTN, hypertension; HPL, hyperlipidemia; DM2, type 2 diabetes; POU, prescription opioid use; CHD, coronary heart disease; MI, myocardial infarction; TS, total stroke, AF, atrial fibrillation.

The results of IVW analyses indicated that genetically determined depression was significantly associated with an increased risk of CHD (meta: OR = 1.136; 95% CI: 1.054,1.224; p = 8.66 × 10^–4^), MI (meta: OR = 1.190; 95% CI, 1.085,1.306; p = 2.32 × 10^–4^), AF (meta: OR = 1.135; 95% CI, 1.059,1.217; p = 3.21 × 10^–4^), and stroke (meta: OR = 1.131; 95% CI, 1.046,1.224; p = 0.002), while no evidence suggested that genetically determined depression was associated with HF risk (Figure 2A).

#### Effects of depression on each mediator

Among the 28 prospective mediators evaluated, four mediators fulfilled the pre-established screening criteria (Figure 1B). IVW results indicated a causal association between genetically determined depression and higher prevalence of type 2 diabetes (OR = 1.158; 95% CI: 1.024,1.309; p = 0.019), hypertension (OR = 1.242; 95% CI: 1.098,1.405; p = 5.62 × 10^–4^), hyperlipidemia (OR = 1.207; 95% CI: 1.046,1.393; p = 5.33 × 10^–4^), and prescription opioid use (OR = 1.375; 95% CI: 1.252,1.510; p = 2.99 × 10^–11^) (Table 4).

**Table 4:** The mediation analysis results of depression on various CVD via mediators.


	NUMBER OF SNPS	β (95%CI))	OR (95%CI)	P

Depression on mediators				

Type 2 diabetes	48	0.147(0.024,0.270)	1.158(1.024,1.309)	0.019

Hypertension	48	0.217(0.094,0.340)	1.242(1.098,1.405)	5.62E–04

Hyperlipidemia	48	0.217(0.094,0.339)	1.242(1.099,1.404)	5.33E–04

Prescription opioid use	48	0.318(0.224,0.412)	1.375(1.252,1.510)	2.99E–11

Type 2 diabetes on CVD				

Coronary heart disease	153	0.131(0.091,0.171)	1.140(1.095,1.187)	1.37E–10

Myocardial infarction	153	0.118(0.076,0.16)	1.125(1.079,1.173)	2.72E–08

Stroke	152	0.095(0.067,0.122)	1.099(1.069,1.130)	2.13E–11

Hypertension on CVD				

Coronary heart disease	138	0.26(0.194,0.325)	1.296(1.214,1.384)	7.36E–15

Myocardial infarction	137	0.24(0.171,0.308)	1.271(1.187,1.361)	7.17E–12

Atrial fibrillation	138	0.184(0.138,0.229)	1.201(1.148,1.258)	3.42E–15

Hyperlipidemia on CVD				

Coronary heart disease	32	0.515(0.371,0.659)	1.673(1.449,1.932)	2.34E–12

Myocardial infarction	32	0.519(0.358,0.680)	1.680(1.430,1.973)	2.69E–10

Stroke	34	0.124(0.063,0.186)	1.133(1.065,1.204)	7.30E–05

Prescription opioid use on CVD				

Stroke	8	0.211(0.039,0.384)	1.236(1.040,1.468)	0.016


Summary MR analysis of the IVW methods for the effect of depression on mediators and the effect of mediators CVD. SNP, Single nucleotide polymorphism; OR, odds ratio; CI, confidence interval; CVD, cardiovascular disease.

#### Effects of each mediator on CVD

Among the four mediators, IVW analysis demonstrated that genetically determined type 2 diabetes and hyperlipidemia increased the risk of CHD (diabetes on CHD: OR = 1.140; 95% CI, 1.095,1.187; p = 1.373 × 10^–10^ and hyperlipidemia on CHD: OR = 1.673; 95% CI, 1.449,1.932; p = 2.34 × 10^–12^), MI (diabetes on MI: OR = 1.125; 95% CI, 1.079,1.173; p = 2.72 × 10^–8^ and hyperlipidemia on MI: OR = 1.680; 95% CI, 1.430,1.973; p = 2.69 × 10^–10^), and stroke (diabetes on stroke: OR = 1.099; 95% CI, 1.069,1.130; p = 2.13 × 10^–11^ and hyperlipidemia on stroke: OR = 1.133; 95% CI, 1.065,1.204; p = 7.30 × 10^–5^). And genetically-determined hypertension is associated with three CVD outcomes, including CHD (OR = 1.296; 95% CI, 1.214,1.384; p = 7.36 × 10^–15^), MI (OR = 1.271; 95% CI, 1.187,1.361; p = 7.17 × 10^–12^), and AF (OR = 1.201; 95% CI, 1.148,1.258; p = 3.42 × 10^–15^). However, genetically-determined prescription opioid use is only related to stroke risk (OR = 1.236; 95% CI, 1.040, 1.468; p = 0.016) (Table 4).

#### Mediating effects of mediators in the association between depression and CVD

Ranked by mediated proportions of four selected mediators, including cardiometabolic risk factors of diabetes, hypertension, hyperlipidemia, and prescription opioid use, the largest causal mediator from depression to CHD was hyperlipidemia (87.06%), followed by hypertension (43.92%) and type 2 diabetes (14.90%). Consistent with the ranking of CHD, the largest causal mediator from depression to MI was hyperlipidemia (64.91%), followed by hypertension (29.87%) and type 2 diabetes (9.77%). The largest causal mediator from depression to stroke was prescription opioid use (54.33%), followed by hyperlipidemia (21.89%) and type 2 diabetes (11.35%). Hypertension is the sole mediator between depression and AF (31.51%) (Figure 2B and Table 5).

**Table 5:** The mediation effect of depression on various CVD via mediators.


EXPOSURE	MEDIATOR	OUTCOME	MEDIATION EFFECT	PROPORTION MEDIATED %

Depression	HPL	CHD	0.111 (0.041, 0.183)	87.06%

Depression	HTN	CHD	0.056 (0.021, 0.091)	43.92%

Depression	DM2	CHD	0.019 (0.002, 0.036)	14.90%

Depression	HPL	MI	0.113 (0.040, 0.186)	64.91%

Depression	HTN	MI	0.052 (0.019, 0.085)	29.87%

Depression	DM2	MI	0.017 (0.002, 0.033)	9.77%

Depression	POU	TS	0.067 (0.009, 0.125)	54.33%

Depression	HPL	TS	0.027 (0.007, 0.047)	21.89%

Depression	DM2	TS	0.014 (0.002, 0.026)	11.35%

Depression	HTN	AF	0.040 (0.015, 0.065)	31.51%


CVD, cardiovascular disease; HTN, hypertension; HPL, hyperlipidemia; DM2, type 2 diabetes; POU, prescription opioid use; CHD, coronary heart disease; MI, myocardial infarction; TS, total stroke, AF, atrial fibrillation.

#### Sensitivity analyses

In the entire analysis, most of the MR analysis models showed consistent direction with the IVW estimates (Tables S3, 5, 6, 8), and genetic IVs used have revealed a diverse range of heterogeneity. In some MR analyses, multiplicative random-effects IVW approach was used, while the fixed-effects IVW approach was employed for others. Moreover, pleiotropy is only observed in the analysis of hypertension on stroke. (Tables S4, 7, 9).

## Discussion

In this study, we conducted observational association analyses between depression and several CVDs in the NHANES dataset. Subsequently, we employed MR analysis to reveal a causal impact of genetically-predicted depression on various CVD risks, including CHD, MI, AF, and stroke. Importantly, we further conducted a comprehensive analysis to examine the potential mediators involved in the relationship between depression and CVD risk. Four out of the 28 modifiable cardiometabolic risk factors were determined as causal mediators. Specifically, type 2 diabetes, hypertension, hyperlipidemia, and prescription opioid use play an important role in the causal pathway from depression to various CVDs. All results are largely consistent between the observational and MR analyses.

Growing evidence has indicated depression is associated with increased risk of hyperlipidemia [21,22], hypertension [23,38], diabetes [22,24], and prescription opioid use [39,40], which are common risk factors for CVD [41,42]. Our results from observational and MR analyses yield consistent conclusions. Among the unselected mediators, we discovered the bidirectional effect between depression and these mediators, including smoking, insomnia, alcohol use disorder, substance abuse, and socioeconomic status, which have been fully studied and proven to be harmful to CVD. Due to the bidirectional relationships between depression and the mediators, which could potentially impact the validity of our results, we refrained from investigating their mediating effect.

Nevertheless, these mediators might still play a regulatory role in the progression of depression leading to CVD and may also be potential confounders in addition to mediators, warranting further research. Furthermore, numerous observational studies have concentrated on establishing the correlation between obesity and depression, and they have indeed confirmed it [20,43]. Nonetheless, an MR analysis has yielded evidence indicating the absence of a direct causal relationship between depression and subsequent changes in body mass [44], consistent with our findings. These findings indicated that obesity may lead to a higher risk of depression, and not vice versa.

A meta-analysis containing 30 prospective cohort studies suggested that depression is independently associated with a significantly increased risk of CHD and MI [8]. Several MR analyses also confirmed the causal harmful impact of depression on CHD and MI [45,46,47]. Our study further solidified the relationship through the meta-MR results of more GWASs for CHD and MI. Furthermore, we observed that the heightened risk of CHD and MI in patients with depression may be mediated by hyperlipidemia, hypertension, and type 2 diabetes. It’s worth highlighting that hyperlipidemia accounts for over half of the mediating effect, contributing to 87.06% for CHD and 64.91% for MI.

A recent Korean cohort study reported that individuals with a prior diagnosis of depression faced a significantly elevated risk of developing new-onset AF, with a hazard ratio (HR) of 1.25 (95% CI, 1.22–1.29). Those experiencing recurrent episodes of depression faced an even greater risk of new-onset AF, with an HR of 1.32 (95% CI, 1.27–1.37) [13]. Meanwhile, findings from a Danish cohort study indicated that individuals using antidepressants had a notably heightened risk of AF [48]. However, two MR studies provided conflicting results [45,47]. Our meta-analysis of MR results indicated that depression increased the risk of AF, with hypertension playing a one-third role in this relationship.

A meta-analysis of 17 prospective studies investigated the temporal relationship between depression and stroke. It specifically focused on initial occurrences of stroke that happened after the baseline evaluations of depression. This meta-analysis conclusively established a positive relationship between depression and the subsequent risk of stroke [49]. A previous MR study has revealed the causal relationship between depression and small vessel stroke [45]. However, other MR studies did not find the causality of depression on total stroke [47] and ischemic stroke [50]. Due to limitations in the available GWAS data, we only studied the causal relationship between depression and total stroke. By using two large-scale GWAS data, we further confirmed the detrimental causal effect of depression on stroke. Hyperlipidemia, type 2 diabetes, and the use of prescription opioids were identified as contributing factors to this relationship, with the use of prescription opioids playing a particularly significant role, accounting for 54.33% of the effect.

Patients diagnosed with depression exhibited an increased risk of HF and a 30-day readmission rate [9,12], and the MR study revealed the causal relationship [45]. Our observational analysis obtained consistent results, but our meta-analysis of MR results did not indicate the causal relationship of depression on HF. The previous MR analysis utilized a single GWAS dataset for HF, and there was a sample overlap between the GWAS datasets for depression and HF, potentially resulting in model overfitting [45]. The findings of previous studies may be influenced by potential confounding factors, such as hyperlipidemia, hypertension, and hyperglycemia.

The heightened susceptibility of individuals with depression to CVD may also be attributed to biological changes, such as inflammation [51], oxidative stress [52], endothelium dysfunction [51], hypothyroidism [53], heightened activity of the sympathoadrenal and pituitary-adrenal axes [54,55,56], increased susceptibility to blood clotting [57], and decreased numbers of circulating endothelial progenitor cells [58], which are known risk factors for CVD. Furthermore, individuals with depression may exhibit diminished adherence to their prescribed medications for these CVDs.

Our study possesses various strengths. Initially, we utilized a comprehensive subtype of CVD data derived from the FinnGen Study and related GWASs, ensuring minimal overlap with exposure, thus reducing the risk of type 1 error. Secondly, we conducted a more detailed and stringent mediation analysis to explore the possible mediating factors in the causal pathway from depression to CVD and excluded interference from reverse causation between depression and these mediators. Thirdly, the reliability of IVW estimates throughout the current investigation was reinforced through the implementation of multiple MR analyses. Inevitably, our study has some limitations that must be acknowledged. Firstly, we only concentrated on cardiometabolic risk factors for which we had available GWAS data. Several potential mediators were not investigated in this study, such as drug compliance. Secondly, the persistent presence of heterogeneity in SNPs may introduce potential bias and impact the reliability of our MR findings. Thirdly, it is worth noting that all study participants belonged to the European ethnic group and originated from high-income economy countries. Further studies of diverse ethnic populations are warranted.

## Conclusion

The findings of our study underscore the causally detrimental influence of depression on CVD. It strengthens the concept that early detection and prevention of depression may mitigate the risk of CVD. Furthermore, we observed a substantial mediating effect of several prevalent cardiometabolic risk factors in the pathway connecting depression to CVD. To decrease the public health burden of CVD among individuals with depression, it may be necessary to implement suitable strategies for managing risk factors such as hyperlipidemia, hypertension, diabetes, and the use of prescription opioids, considering their potential roles in mediating the pathway from depression to CVD.

## Data Accessibility Statement

All the data used in the present study had been publicly available. The original contributions presented in the study are included in the article/Supplementary Material. Further inquiries can be directed to the corresponding author.

## Additional File

The additional file for this article can be found as follows:

10.5334/gh.1302.s1 Supplementary material. Tables S1–S9, Figures S1–S2.
