# Supplementary material for: Heartache and Heartbreak: An Observational and Mendelian Randomization Study
Source: Glob Heart. 2024 Feb 16;19(1):19. doi: 10.5334/gh.1302 (PMC10870952; doi:10.5334/gh.1302)
Supplement: Supplementary material. — Tables S1–S9, Figures S1–S2. [file gh-19-1-1302-s1.pdf]

Supplementary Material

Table S1 Characteristics of SNPs used as instrumental variables for depression 1
Table S2. Detailed information on data sources 2
Table S3. MR estimating the associations of depression with cardiovascular diseases 4
Table S4. MR heterogeneity test and directional pleiotropy test (MR Egger) of the associations of depression on cardiovascular diseases 5
Table S5. MR estimating the associations of depression on mediator 5
Table S6. MR estimating the associations of mediator on depression 6
Table S7. MR heterogeneity test and directional pleiotropy test (MR Egger) of the associations between depression and mediators 6
Table S8. MR estimating the associations of each mediator on cardiovascular diseases 7
Table S9. MR heterogeneity test and directional pleiotropy test (MR Egger) of the associations of each mediator on cardiovascular diseases 8
Figure S1. Flowchart of the NHANES study population. 10
Figure S2 Overview of two-step mediation analysis of depression on cardiovascular diseases via potential mediators. 10

Abbreviation: MR, Mendelian randomization; OR, Odds ratio; CI, confidence interval; IVW, inverse variance weighted; PRESSO, pleiotropy residual sum and outlier; SNP, single nucleotide polymorphism; CARDIoGRAMplusC4D, Coronary Artery Disease Genome wide Replication and Meta-analysis (CARDIoGRAM) plus the Coronary Artery Disease (C4D) Genetics consortium; HERMES, Heart Failure Molecular Epidemiology for Therapeutic Targets.

Table S1 Characteristics of SNPs used as instrumental variables for depression

SNP	chr	pos	effect_allele	other_allele	beta	se	pval	F-statistics
rs7551758	1	52274078	G	T	0.028	0.004	5.11E-11	43.31
rs2568958	1	72765116	A	G	0.038	0.004	2.90E-18	75.37
rs10913112	1	175913828	T	C	-0.026	0.005	4.53E-09	33.90
rs17641524	1	197704717	T	C	-0.030	0.005	1.50E-08	32.04
rs354155	1	49675276	C	G	-0.045	0.008	1.75E-09	35.84
rs7538938	1	67132262	C	T	0.025	0.004	7.29E-09	34.07
rs4141983	1	18122009	C	T	-0.026	0.005	9.69E-09	32.94
rs2111592	2	208049581	A	G	0.026	0.005	1.35E-08	32.69
rs72948506	2	212618440	A	G	0.027	0.005	1.71E-08	31.79
rs843812	3	61255413	A	G	0.025	0.004	1.41E-08	31.77
rs9831648	3	49214303	T	G	-0.029	0.005	1.59E-08	31.53
rs66511648	3	117515519	C	T	0.030	0.005	6.03E-10	38.29
rs76954012	3	115977242	A	T	0.041	0.007	2.41E-08	31.00
rs30266	5	103972357	A	G	0.037	0.005	1.43E-15	63.31
rs247910	5	87630769	G	A	0.024	0.004	4.71E-08	30.38

rs7725715	5	164487555	A	G	0.029	0.004	1.61E-11	45.48
rs150186873	6	27182377	C	A	0.070	0.012	4.51E-09	34.42
rs2232423	6	28366151	G	A	-0.062	0.007	1.14E-18	78.45
rs9364755	6	165117329	G	A	0.028	0.005	3.49E-08	30.79
rs2214123	6	67000001	G	A	-0.026	0.005	8.56E-09	33.64
rs2876520	6	142996618	G	C	0.026	0.004	2.24E-09	36.56
rs2522831	7	82448100	C	T	0.024	0.004	2.11E-08	31.15
rs4730387	7	109100414	A	T	0.024	0.004	4.12E-08	30.63
rs150346963	7	117625599	T	C	0.028	0.004	1.16E-10	41.37
rs3807865	7	12250402	A	G	0.031	0.004	1.09E-12	49.64
rs10235664	7	2086814	C	T	-0.027	0.005	4.68E-08	30.36
rs59082935	7	38724868	T	C	0.036	0.007	3.07E-08	30.25
rs62535714	9	37182655	A	G	0.034	0.006	4.69E-09	34.16
rs1931388	9	11203149	G	A	-0.030	0.004	1.68E-11	44.95
rs59283172	9	25232978	A	G	-0.039	0.007	2.41E-08	31.04
rs2418449	9	119731359	C	T	-0.028	0.005	4.25E-09	34.27
rs1021363	10	106610839	G	A	-0.030	0.005	2.29E-11	44.44
rs198457	11	61471678	T	C	-0.032	0.006	1.90E-08	31.64
rs4497414	11	88756779	C	T	0.029	0.004	2.93E-11	43.74
rs4936276	11	113365141	C	G	0.028	0.004	3.57E-10	39.92
rs61914045	12	52352301	A	G	0.031	0.005	7.96E-09	32.74
rs9529218	13	31790053	T	C	-0.034	0.005	2.23E-10	39.64
rs9536381	13	53860655	T	C	0.026	0.005	2.62E-08	30.73
rs508502	13	80921519	T	C	-0.026	0.005	3.56E-08	30.25
rs1950829	14	42097937	G	A	-0.030	0.004	4.74E-12	47.71
rs754287	14	103997525	A	T	-0.029	0.005	1.31E-10	41.24
rs7152906	14	75125540	C	T	0.026	0.004	1.87E-09	36.00
rs28541419	15	88945878	G	C	-0.029	0.005	1.76E-08	31.53
rs12919291	16	13800430	C	G	0.033	0.006	3.09E-09	35.35
rs4799949	18	35155910	T	C	-0.029	0.005	1.40E-10	40.29
rs12967143	18	53099012	C	G	-0.035	0.005	2.53E-13	53.88
rs7241572	18	77580712	A	G	0.032	0.005	2.43E-09	35.78
rs1367635	18	50861409	C	T	0.025	0.004	4.35E-09	34.62
rs13037326	20	44692598	T	C	0.031	0.005	2.40E-10	40.02

F-statistics = beta/se

Table S2. Detailed information on data sources

Phenotype	Consortium	Cases / Controls	Ancestry	Year	PubMed ID
**Exposure**					
Major depression	PGC	170,756/329,443	European	2019	30718901
**Outcome**					
Coronary heart disease	CARDIoGRAMplusC4D	60,801/123,504	77% European	2015	26343387
	FinnGen	43,518/333,759	European	2023	NA
Myocardial infarction	CARDIoGRAMplusC4D	43,676/128,199	77% European	2015	26343387

	FinnGen	24,185/313,400	European	2023	NA
Atrial fibrillation	Nielsen et al	60,620/ 970,216	European	2018	30061737
	FinnGen	45,766/191,924	European	2023	NA
Heart failure	HERMES	47,309/ 930,014	European	2020	31919418
	FinnGen	26,872/349,361	European	2023	NA
Total stroke	MEGASTROKE	40,585/406,111	European	2018	29531354
	FinnGen	39,818/271,817	European	2023	NA
**Selected mediator**					
Prescription opioid use	FinnGen	57,823/319,454	European	2023	NA
Type 2 diabetes	FinnGen	57,698/308,252	European	2023	NA
Hypertension	FinnGen	111,581/265,626	European	2023	NA
Hyperlipidemia	FinnGen	37,742/324,150	European	2023	NA
**Excluded mediator**					
Smoking initiation	GSCAN	311,629/321,173	European	2019	30643251
Age of smoking	GSCAN	341,427	European	2019	30643251
Smoking heaviness	GSCAN	249,752	European	2019	30643251
Alcohol drinking	GSCAN	335,394	European	2019	30643251
alcohol use disorder	FinnGen	15715/36156	European	2023	NA
substance abuse	FinnGen	23194/277526	European	2023	NA
Insomnia complaints	Hammerschlag et al	12,863/19,521	European	2017	28604731
Insomnia	UK biobank	336,965	European	2018	NA
Daytime nap	Dashti et al	452,633	European	2021	33568662
Diastolic blood pressure	ICBP	757,601	European	2018	30224653
Systolic blood pressure	ICBP	757,601	European	2018	30224653
Total cholesterol in HDL	Kettunen et al	21,555	European	2016	27005778
Apolipoprotein A-I	Kettunen et al	20,687	European	2016	27005778
Total cholesterol in LDL	Kettunen et al	21,559	European	2016	27005778
Apolipoprotein B	Kettunen et al	20,690	European	2016	27005778
Serum total cholesterol	Kettunen et al	21,491	European	2016	27005778
Serum total triglycerides	Kettunen et al	21,545	European	2016	27005778
Education	SSGAC	766,345	European	2018	30038396
Household income before tax	UK Biobank	397,751	European	2018	NA
Townsend deprivation index	UK Biobank	462,464	European	2018	NA
MVPA	UK Biobank	377,234	European	2018	29899525
Watching television	UK Biobank	437,887	European	2018	NA
obesity	FinnGen	21375/355786	European	2023	NA
body mass index	FinnGen	266130	European	2023	NA

PGC, Psychiatric Genomic Consortium; GSCAN, GWAS & Sequencing Consortium of Alcohol and Nicotine use; GWAS, genome-wide association study; CARDIoGRAMplusC4D, Coronary Artery Disease Genome wide Replication and Meta-analysis (CARDIoGRAM) plus the Coronary Artery Disease (C4D) Genetics consortium; HERMES, Heart Failure Molecular Epidemiology for Therapeutic Targets; ICBP, International Consortium of Blood Pressure; SSGAC, Science Genetic Association Consortium ; LDL-C, low-density lipoprotein cholesterol; HDL-C, high-density lipoprotein cholesterol; MVPA, moderate to vigorous physical activity; NA, not available.

Table S3. MR estimating the associations of depression with cardiovascular diseases

Outcome	Method	No. of SNPs	OR (95% CI)	P value
Coronary heart disease (CARDIoGRAMplusC4D)	IVW	49	1.147(1.038,1.268)	0.007
	Weighted median	49	1.212(1.054,1.394)	0.007
	MR Egger	49	1.126(0.640,1.980)	0.683
	MR PRESSO	0*	1.147(1.042,1.262)	0.007
Coronary heart disease (FinnGen)	IVW	48	1.122(1.002,1.257)	0.047
	Weighted median	48	1.147(0.998,1.317)	0.053
	MR Egger	48	0.660(0.340,1.279)	0.224
	MR PRESSO	1*	1.098(0.984,1.224)	0.101
Myocardial infarction (CARDIoGRAMplusC4D)	IVW	49	1.209(1.082,1.350)	0.001
	Weighted median	49	1.210(1.028,1.424)	0.022
	MR Egger	49	1.127(0.603,2.105)	0.709
	MR PRESSO	0*	1.209(1.083,1.350)	0.001
Myocardial infarction (FinnGen)	IVW	48	1.147(0.968,1.359)	0.113
	Weighted median	48	1.114(0.924,1.344)	0.257
	MR Egger	48	0.716(0.262,1.954)	0.517
	MR PRESSO	0*	1.141(0.972,1.340)	0.114
Atrial fibrillation (Six studies)	IVW	49	1.089(0.994,1.193)	0.067
	Weighted median	49	1.106(0.994,1.230)	0.064
	MR Egger	49	1.605(0.983,2.620)	0.064
	MR PRESSO	0*	1.089(0.994,1.193)	0.073
Atrial fibrillation (FinnGen)	IVW	48	1.201(1.080,1.335)	7.18E-04
	Weighted median	48	1.189(1.019,1.389)	0.028
	MR Egger	48	1.457(0.737,2.879)	0.285
	MR PRESSO	0*	1.201(1.071,1.346)	0.003
Heart failure (HERMES)	IVW	49	1.185(1.071,1.312)	0.001
	Weighted median	49	1.133(1.000,1.285)	0.050
	MR Egger	49	1.165(0.654,2.074)	0.607
	MR PRESSO	0*	1.185(1.071,1.312)	0.002
Heart failure (FinnGen)	IVW	48	0.951(0.857,1.054)	0.339
	Weighted median	48	0.915(0.782,1.071)	0.270
	MR Egger	48	0.652(0.326,1.302)	0.231
	MR PRESSO	0*	0.951(0.845,1.069)	0.402
Stroke (MEGASTROKE)	IVW	49	1.075(0.953,1.213)	0.237
	Weighted median	49	0.969(0.834,1.125)	0.679
	MR Egger	49	1.189(0.607,2.331)	0.616
	MR PRESSO	1*	1.044(0.936,1.164)	0.447
Stroke (FinnGen)	IVW	48	1.175(1.059,1.304)	0.002
	Weighted median	48	1.164(1.003,1.351)	0.046
	MR Egger	48	1.279(0.670,2.442)	0.459
	MR PRESSO	0*	1.175(1.055,1.309)	0.005

*the number of outlier detected

Table S4. MR heterogeneity test and directional pleiotropy test (MR Egger) of the associations of depression on cardiovascular diseases

Outcome	Method	Q statistic	Q df	Q p-value	Egger intercept	SE	P value
Coronary heart disease (CARDIoGRAMplusC4D)	IVW	43.673	47	0.611			
	MR Egger	43.677	48	0.650	0.001	0.009	0.948
Coronary heart disease (FinnGen)	IVW	75.089	47	0.004			
	MR Egger	79.246	48	0.002	0.016	0.010	0.117
Myocardial infarction (CARDIoGRAMplusC4D)	IVW	47.544	47	0.450			
	MR Egger	47.594	48	0.489	0.002	0.010	0.824
Myocardial infarction (FinnGen)	IVW	101.318	47	4.87E-06			
	MR Egger	103.237	48	4.26E-06	0.014	0.015	0.355
Atrial fibrillation (Six studies)	IVW	74.908	47	0.006			
	MR Egger	78.883	48	0.003	-0.012	0.007	0.121
Atrial fibrillation (FinnGen)	IVW	54.495	47	0.183			
	MR Egger	54.872	48	0.201	-0.006	0.010	0.575
Heart failure (HERMES)	IVW	69.452	47	0.018			
	MR Egger	69.457	48	0.023	0.001	0.009	0.952
Heart failure (FinnGen)	IVW	58.766	47	0.098			
	MR Egger	60.271	48	0.093	0.011	0.010	0.283
Stroke (MEGASTROKE)	IVW	72.656	47	0.010			
	MR Egger	72.793	48	0.012	-0.003	0.010	0.265
Stroke (FinnGen)	IVW	50.741	47	0.292			
	MR Egger	50.816	48	0.326	-0.003	0.010	0.795

Table S5. MR estimating the associations of depression on mediator

Mediator	Method	No. Of SNPs	β (95% CI)	OR (95% CI)	P value
Hypertension (FinnGen)	IVW	48	0.217(0.094,0.34)	1.242(1.098,1.405)	5.62E-04
	Weighted median	48	0.173(0.06,0.286)	1.189(1.061,1.331)	0.003
	MR Egger	48	-0.042(-0.775,0.692)	0.959(0.461,1.998)	0.912
	MR PRESSO	5*	0.163(0.057,0.269)	1.177(1.059,1.308)	0.004
Type 2 diabetes (FinnGen)	IVW	48	0.147(0.024,0.270)	1.158(1.024,1.309)	0.019
	Weighted median	48	0.159(0.030,0.288)	1.172(1.030,1.334)	0.016
	MR Egger	48	0.536(-0.191,1.262)	1.709(0.826,3.533)	0.155
	MR PRESSO	2*	0.144(0.030,0.259)	1.155(1.030,1.296)	0.018
Hyperlipidemian (FinnGen)	IVW	48	0.217(0.094,0.339)	1.242(1.099,1.404)	5.33E-04
	Weighted median	48	0.194(0.052,0.336)	1.215(1.054,1.400)	0.007
	MR Egger	48	-0.035(-0.766,0.695)	0.965(0.465,2.004)	0.925
	MR PRESSO	0*	0.217(0.094,0.339)	1.242(1.099,1.404)	0.001
Prescription opioid use	IVW	48	0.318(0.224,0.412)	1.375(1.252,1.510)	2.99E-11

(FinnGen)	Weighted median	48	0.319(0.211,0.427)	1.376(1.235,1.532)	6.28E-09
	MR Egger	48	0.301(-0.262,0.864)	1.352(0.770,2.374)	0.300
	MR PRESSO	1*	0.337(0.248,0.426)	1.401(1.282,1.532)	2.19E-09

*the number of outlier detected

Table S6. MR estimating the associations of mediator on depression

Mediator	Method	No. Of SNPs	β (95% CI)	OR (95% CI)	P value
Hypertension (FinnGen)	IVW	124	0.021(-0.005,0.046)	1.021(0.995,1.047)	0.110
	Weighted median	124	0.011(-0.019,0.040)	1.011(0.982,1.041)	0.470
	MR Egger	124	-0.002(-0.086,0.081)	0.998(0.918,1.084)	0.956
	MR PRESSO	4*	0.016(-0.007,0.039)	1.016(0.993,1.039)	0.180
Type 2 diabetes (FinnGen)	IVW	134	0.003(-0.015,0.022)	1.003(0.985,1.022)	0.741
	Weighted median	134	-0.004(-0.027,0.018)	0.996(0.974,1.018)	0.693
	MR Egger	134	-0.014(-0.057,0.028)	0.986(0.945,1.029)	0.514
	MR PRESSO	3*	-0.002(-0.019,0.015)	0.998(0.981,1.015)	0.819
Hyperlipidemian (FinnGen)	IVW	32	-0.006(-0.024,0.012)	0.994(0.976,1.012)	0.524
	Weighted median	32	-0.010(-0.037,0.017)	0.990(0.964,1.017)	0.455
	MR Egger	32	-0.025(-0.059,0.008)	0.975(0.943,1.008)	0.149
	MR PRESSO	0*	-0.006(-0.025,0.013)	0.994(0.975,1.013)	0.555
Prescription opioid use (FinnGen)	IVW	8	0.117(-0.007,0.240)	1.124(0.993,1.271)	0.064
	Weighted median	8	0.073(-0.037,0.183)	1.075(0.963,1.201)	0.196
	MR Egger	8	-0.407(-1.267,0.452)	0.665(0.282,1.571)	0.389
	MR PRESSO	1*	0.084(-0.020,0.188)	1.088(0.980,1.207)	0.164

*the number of outlier detected

Table S7. MR heterogeneity test and directional pleiotropy test (MR Egger) of the associations between depression and mediators

	Method	Q statistic	Q df	Q p-value	Egger intercept	SE	P value
Depression on Hypertension	IVW	160.778	47	1.22E-14			
	MR Egger	162.492	48	1.24E-14	0.008	0.011	0.487
Depression on Type 2 diabetes	IVW	127.535	47	1.37E-09			
	MR Egger	130.676	48	8.21E-10	-0.012	0.011	0.293
Depression on Hyperlipidemian	IVW	93.066	47	4.96E-05			
	MR Egger	94.018	48	5.60E-05	0.008	0.011	0.496
Depression on Prescription opioid use	IVW	84.777	47	4.35E-04			
	MR Egger	84.783	48	6.06E-04	0.001	0.009	0.953
Hypertension on Depression	IVW	255.730	122	1.67E-11			
	MR Egger	256.413	123	2.04E-11	0.001	0.002	0.569
Type 2 diabetes on Depression	IVW	261.454	132	1.46E-10			
	MR Egger	263.025	133	1.40E-10	0.001	0.001	0.375
Hyperlipidemian on	IVW	33.274	30	0.311			

Depression	MR Egger	35.387	31	0.269	0.003	0.002	0.178
Prescription opioid use on Depression	IVW	13.677	6	0.033			
	MR Egger	16.999	7	0.017	0.022	0.019	0.273

Table S8. MR estimating the associations of each mediator on cardiovascular diseases

Mediator on Outcome	Method	No. Of SNPs	β (95% CI)	OR (95% CI)	P value
Hypertension on Coronary heart disease (CARDIoGRAMplusC4D)	IVW	138	0.260(0.194,0.325）	1.296(1.214,1.384）	7.36E-15
	Weighted median	138	0.207(0.144,0.269）	1.230(1.155,1.308）	7.21E-11
	MR Egger	138	0.438(0.227,0.650）	1.550(1.255,1.916）	8.23E-05
	MR PRESSO	6*	0.255(0.205,0.306）	1.291(1.227,1.358）	1.84E-17
Hypertension on Myocardial infarction (CARDIoGRAMplusC4D)	IVW	137	0.240(0.171,0.308）	1.271(1.187,1.361）	7.17E-12
	Weighted median	137	0.199(0.128,0.270）	1.220(1.137,1.310）	3.42E-08
	MR Egger	137	0.370(0.146,0.594）	1.448(1.157,1.812）	0.002
	MR PRESSO	6*	0.237(0.183,0.291）	1.267(1.200,1.338）	3.21E-14
Hypertension on Atrial fibrillation (Six studies)	IVW	138	0.184(0.138,0.229）	1.201(1.148,1.258）	3.42E-15
	Weighted median	138	0.166(0.120,0.212）	1.181(1.128,1.236）	1.24E-12
	MR Egger	138	0.170(0.022,0.319）	1.186(1.022,1.376）	0.026
	MR PRESSO	9*	0.185(0.148,0.222）	1.203(1.160,1.248）	1.47E-17
Hypertension on Stroke (MEGASTROKE)	IVW	137	0.286(0.239,0.334）	1.331(1.270,1.396）	2.30E-32
	Weighted median	137	0.295(0.236,0.353）	1.342(1.266,1.423）	4.41E-23
	MR Egger	137	0.438(0.285,0.592）	1.550(1.330,1.807）	1.11E-07
	MR PRESSO	2*	0.270(0.225,0.314）	1.309(1.252,1.369）	9.96E-23
Type 2 diabetes on Coronary heart disease (CARDIoGRAMplusC4D)	IVW	153	0.131(0.091,0.171）	1.140(1.095,1.187）	1.37E-10
	Weighted median	153	0.130(0.073,0.187）	1.139(1.076,1.206）	8.19E-06
	MR Egger	153	0.089(-0.001,0.178）	1.093(0.999,1.195）	0.053
	MR PRESSO	5*	0.132(0.098,0.165）	1.141(1.103,1.180）	1.47E-12
Type 2 diabetes on Myocardial infarction (CARDIoGRAMplusC4D)	IVW	153	0.118(0.076,0.160）	1.125(1.079,1.173）	2.72E-08
	Weighted median	153	0.100(0.041,0.160）	1.105(1.041,1.173）	9.79E-04
	MR Egger	153	0.071(-0.021,0.163）	1.074(0.979,1.177）	0.132
	MR PRESSO	4*	0.113(0.078,0.149）	1.120(1.081,1.161）	3.49E-09
Type 2 diabetes on Atrial fibrillation (Six studies)	IVW	159	0.010(-0.022,0.041）	1.010(0.979,1.042）	0.541
	Weighted median	159	-0.019(-0.055,0.017）	0.981(0.946,1.017）	0.293
	MR Egger	159	-0.053(-0.122,0.017）	0.949(0.885,1.017）	0.139
	MR PRESSO	7*	0.011(-0.017,0.040）	1.012(0.983,1.040）	0.426
Type 2 diabetes on Stroke (MEGASTROKE)	IVW	152	0.095(0.067,0.122）	1.099(1.069,1.130）	2.13E-11
	Weighted median	152	0.102(0.052,0.152）	1.108(1.054,1.164）	6.18E-05
	MR Egger	152	0.082(0.016,0.148）	1.086(1.017,1.160）	0.016
	MR PRESSO	0*	0.095(0.066,0.123）	1.099(1.068,1.131）	1.77E-09
Hyperlipidemian on Coronary heart disease (CARDIoGRAMplusC4D)	IVW	32	0.515(0.371,0.659)	1.673(1.449,1.932)	2.34E-12
	Weighted median	32	0.355(0.273,0.437)	1.426(1.314,1.548)	2.09E-17
	MR Egger	32	0.435(0.169,0.701)	1.545(1.184,2.016)	0.003

	MR PRESSO	8*	0.503(0.407,0.599)	1.654(1.503,1.821)	4.57E-10
Hyperlipidemian on Myocardial infarction (CARDIoGRAMplusC4D)	IVW	32	0.519(0.358,0.680)	1.680(1.430,1.973)	2.69E-10
	Weighted median	32	0.309(0.224,0.394)	1.362(1.252,1.483)	8.77E-13
	MR Egger	32	0.381(0.080,0.681)	1.463(1.084,1.976)	0.019
	MR PRESSO	4*	0.479(0.374,0.584)	1.615(1.454,1.793)	1.39E-09
Hyperlipidemian on Atrial fibrillation (Six studies)	IVW	39	0.041(-0.009,0.09)	1.041(0.991,1.094)	0.107
	Weighted median	39	0.019(-0.028,0.065)	1.019(0.973,1.067)	0.431
	MR Egger	39	0.016(-0.074,0.105)	1.016(0.929,1.111)	0.731
	MR PRESSO	3*	0.028(-0.011,0.067)	1.029(0.989,1.070)	0.169
Hyperlipidemian on Stroke (MEGASTROKE)	IVW	34	0.124(0.063,0.186)	1.133(1.065,1.204)	7.30E-05
	Weighted median	34	0.102(0.029,0.174)	1.107(1.030,1.190)	0.006
	MR Egger	34	0.146(0.033,0.259)	1.157(1.034,1.295)	0.016
	MR PRESSO	2*	0.099(0.046,0.151)	1.104(1.048,1.163)	8.27E-04
Prescription opioid use on Coronary heart disease (CARDIoGRAMplusC4D)	IVW	8	-0.086(-0.561,0.390）	0.918(0.571,1.477）	0.724
	Weighted median	8	-0.038(-0.297,0.220）	0.962(0.743,1.246）	0.771
	MR Egger	8	-3.080(-6.121,-0.038）	0.046(0.002,0.963）	0.094
	MR PRESSO	2*	-0.012(-0.206,0.182）	0.988(0.814,1.199）	0.907
Prescription opioid use on Myocardial infarction (CARDIoGRAMplusC4D)	IVW	8	-0.030(-0.560,0.500）	0.971(0.571,1.649）	0.912
	Weighted median	8	-0.060(-0.354,0.234）	0.941(0.702,1.263）	0.687
	MR Egger	8	-3.406(-6.790,-0.019）	0.033(0.001,0.981）	0.096
	MR PRESSO	3*	-0.051(-0.188,0.086）	0.950(0.828,1.090）	0.504
Prescription opioid use on Atrial fibrillation (Six studies)	IVW	8	0.092(-0.029,0.214）	1.097(0.971,1.238）	0.136
	Weighted median	8	0.152(-0.006,0.309）	1.164(0.994,1.363）	0.059
	MR Egger	8	0.645(-0.224,1.513）	1.905(0.799,4.542）	0.196
	MR PRESSO	0*	0.092(-0.002,0.186）	1.097(0.998,1.205）	0.090
Prescription opioid use on Stroke (MEGASTROKE)	IVW	8	0.211(0.039,0.384）	1.236(1.040,1.468）	0.016
	Weighted median	8	0.307(0.071,0.544）	1.360(1.073,1.722）	0.011
	MR Egger	8	-0.638(-1.867,0.591）	0.528(0.155,1.805）	0.348
	MR PRESSO	0*	0.211(0.031,0.392）	1.236(1.031,1.481）	0.056

*the number of outlier detected

Table S9. MR heterogeneity test and directional pleiotropy test (MR Egger) of the associations of each mediator on cardiovascular diseases

Mediator on Outcome	Method	Q statistic	Q df	Q p-value	Egger intercept	SE	P value
Hypertension on Coronary heart disease	IVW	430.790	136	2.40E-32			
	MR Egger	440.374	137	1.56E-33	-0.010	0.006	0.084
Hypertension on Myocardial infarction	IVW	383.305	135	1.23E-25			
	MR Egger	387.380	136	5.47E-26	-0.007	0.006	0.233
Hypertension on Atrial fibrillation	IVW	417.034	136	2.69E-30			
	MR Egger	417.137	137	4.57E-30	0.001	0.004	0.855
Hypertension on Stroke	IVW	224.766	135	1.96E-06			
	MR Egger	231.720	136	5.86E-07	-0.008	0.004	0.043

Type 2 diabetes on Coronary heart disease	IVW	315.342	151	1.19E-13			
	MR Egger	317.627	152	9.40E-14	0.003	0.003	0.297
Type 2 diabetes on Myocardial infarction	IVW	276.768	151	1.92E-09			
	MR Egger	279.064	152	1.53E-09	0.004	0.003	0.265
Type 2 diabetes on Atrial fibrillation	IVW	401.107	157	2.67E-23			
	MR Egger	411.150	158	1.92E-24	0.005	0.002	0.049
Type 2 diabetes on Stroke	IVW	164.681	150	0.195			
	MR Egger	164.861	151	0.208	0.001	0.002	0.686
Hyperlipidemian on Coronary heart disease	IVW	318.793	30	5.12E-50			
	MR Egger	324.018	31	1.56E-50	0.010	0.015	0.489
Hyperlipidemian on Myocardial infarction	IVW	322.022	30	1.17E-50			
	MR Egger	334.251	31	1.47E-52	0.018	0.017	0.294
Hyperlipidemian on Atrial fibrillation	IVW	116.358	37	3.91E-10			
	MR Egger	117.718	38	4.39E-10	0.003	0.005	0.515
Hyperlipidemian on Stroke	IVW	74.645	32	2.92E-05			
	MR Egger	75.111	33	3.99E-05	-0.003	0.006	0.658
Prescription opioid use on Coronary heart disease	IVW	31.621	6	1.93E-05			
	MR Egger	51.584	7	7.05E-09	0.126	0.065	0.100
Prescription opioid use on Myocardial infarction	IVW	31.556	6	1.98E-05			
	MR Egger	51.988	7	5.87E-09	0.142	0.072	0.096
Prescription opioid use on Atrial fibrillation	IVW	3.202	6	0.783			
	MR Egger	4.786	7	0.686	-0.024	0.019	0.255
Prescription opioid use on Stroke	IVW	5.840	6	0.441			
	MR Egger	7.714	7	0.358	0.036	0.027	0.220

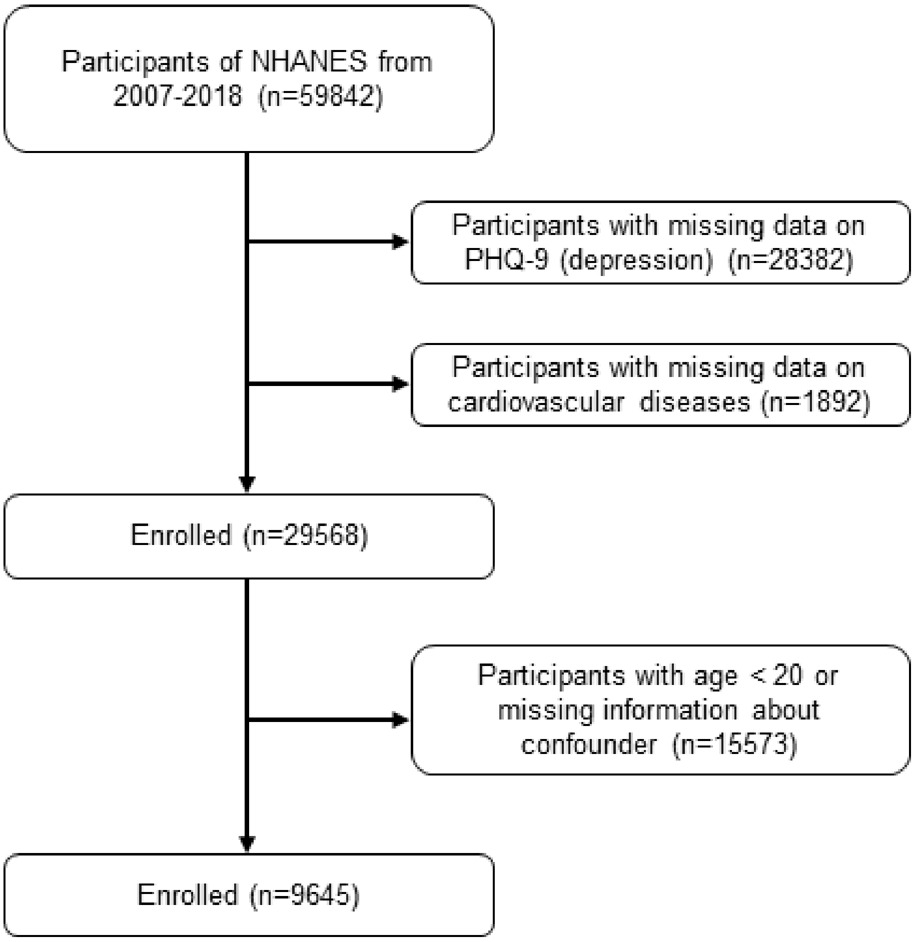

Figure S1. Flowchart of the NHANES study population.

NHANES, National Health and Nutrition Examination Survey; PHQ-9, 9-item Patient Health Questionnaire.

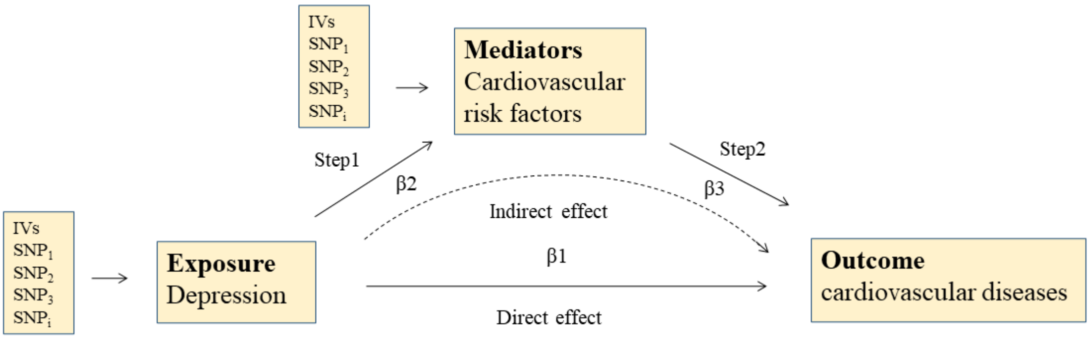

Figure S2 Overview of two-step mediation analysis of depression on cardiovascular diseases via potential mediators.

IVs, instrumental variables. Direct effect =β1–β2*β3; Indirect effect = β2*β3. Proportion mediated = β2*β3/β1
